# Supplementary material for: “Candidatus Siderophilus nitratireducens”: a putative nap-dependent nitrate-reducing iron oxidizer within the new order Siderophiliales
Source: ISME Commun. 2024 Jan 20;4(1):ycae008. doi: 10.1093/ismeco/ycae008 (PMC10993476; doi:10.1093/ismeco/ycae008)
Supplement: Supplementary_Information_ycae008 [file supplementary_information_ycae008.docx]

Supplementary Information for

“*Candidatus* Siderophilus nitratireducens”: a putative *nap*-dependent nitrate-reducing iron oxidizer within the new order Siderophiliales

Francesc Corbera-Rubio^a^, Gerben R. Stouten^a^, Jantinus Bruins^b^, Simon F. Dost^c^, Alexander Y. Merkel^d^ , Simon Müller^a^, Mark C. M. van Loosdrecht^a^, Doris van Halem^a^, Michele Laureni^a,*^

* corresponding author: [m.laureni@tudelft.nl](mailto:m.laureni@tudelft.nl)

^a^ Delft University of Technology, Delft, the Netherlands
^b^ WLN, Glimmen, the Netherlands
^c^ WMD Water company Drenthe, Assen, the Netherlands
^d^ Winogradsky Institute of Microbiology, Research Center of Biotechnology, Russian Academy of Sciences, Moscow, Russia

# Pilot-scale filter and groundwater characteristics


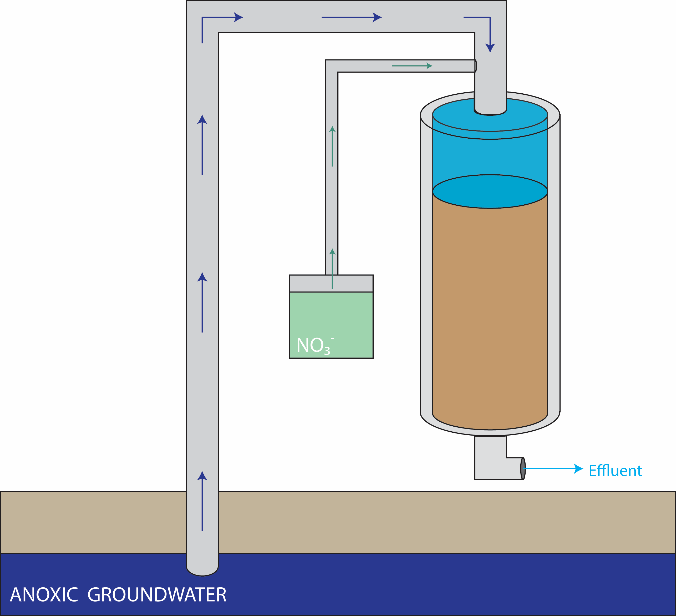


Figure S1. Scheme of the pilot-scale set-up. Anoxic groundwater containing iron and nitrate was fed into a filter filled with granular activated carbon. Nitrate was added manually into the groundwater prior to the filter in the second phase of the experiment.

Table S1. Operational and design parameters of the pilot-scale filter.

| **Parameter** | **Units** | **Value** |
| --- | --- | --- |
| **Bed height** | m | 1.34 |
| **Filter area** | m^2^ | 0.0078 |
| **Empty bed contact** | min | 21 |
| **Filtration velocity** | m/h | 3.8 |

Figure S2. Pilot-scale rapid sand filter filled with granular activated carbon and fed with anoxic groundwater.

# Continuous operation of pilot-scale filter


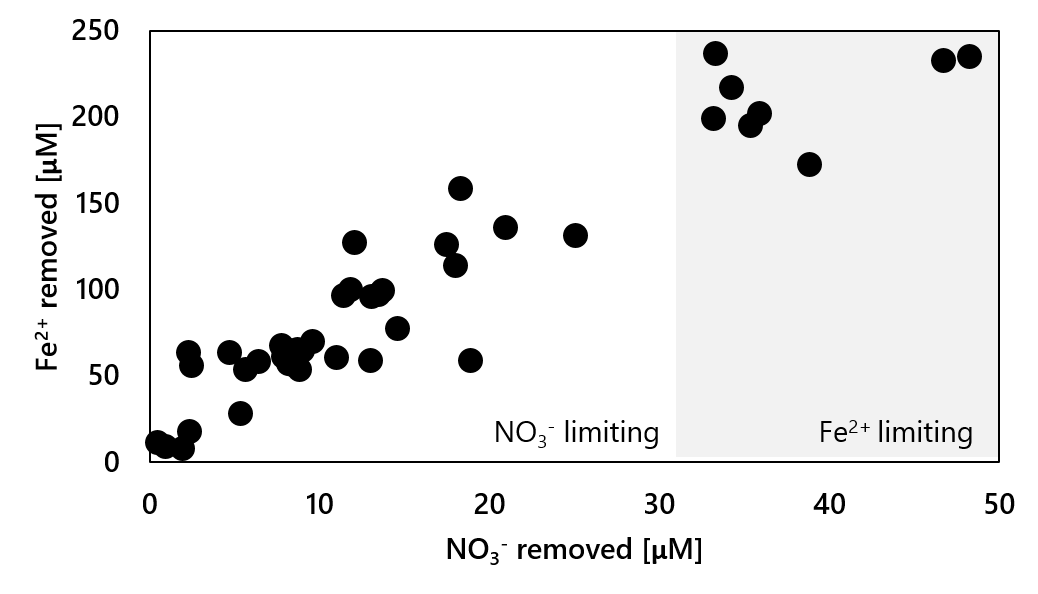


Figure S 3. Simultaneous and proportional NO3- and Fe2+ removals in the groundwater-fed pilot-scale filter during the 120 days of continuous operation, regardless of limiting nutrient. The groundwater Fe2+ concentration was constant throughout the experiment (236 ± 4 µM). NO3- was dosed in the influent to step-wise increase the natural groundwater concentration from 8.1 ± 2.1 to 20.2 ± 2.4 µM (NO3- limitation), and up to 83.8 ± 0.6 µM (Fe2+ limitation).

# Metagenomic analysis

Table S1. Main metabolic pathways and enzymes in the genome of “Ca. Siderophilus Nitratireducens”. ORF were predicted using Prodigal v2.6.3. (1), and functional annotation was carried out with GhostKoala v.2.2 (ac. March 2022) (2) and the Prokaryotic Genome Annotation Pipeline v6.1. (3). The annotation of iron oxidation genes was refined with FeGenie (4). Predicted genes were aligned against the Non-redundant proteins sequences (nr) database from NCBI using blastp and accepted if coverage >70% and identity > 35% (5).

[See Table S1 as attached separate Excel file]

# Solid identification with Mössbauer spectroscopy

## Materials and methods

Transmission ^57^Fe Mössbauer spectrum was collected at 4.2 K with a sinusoidal velocity spectrometer using a ^57^Co(Rh) source. Velocity calibration was carried out using an α-Fe foil at room temperature. The source and the absorbing sample were kept at the same temperature during the measurement. The Mössbauer spectrum was fitted using the Mosswinn 4.0 program (6).

## Results

*Figure S4. Mossbauer spectra obtained at 4.2 K*

*with iron oxide minerals.*

Table S2. The Mössbauer fitted parameters of iron oxide minerals

| *Sample* | *IS*  *(mm·s^-1^)* | *QS*  *(mm·s^-1^)* | *Hyperfine field (T)* | *Γ*  *(mm·s^-1^)* | *Phase* | *Spectral contribution (%)* |
| --- | --- | --- | --- | --- | --- | --- |
| B. NRFO | 0.32  0.35 | -0.04  -0.05 | 43.1^*^  48.7 | 0.56  0.65 | Fe^3+^ (Ferrihydrite^a^)  Fe^3+^ (Ferrihydrite^b^) | 43  57 |

*Experimental uncertainties: Isomer shift: I.S. ± 0.01 mm s-1; Quadrupole splitting: Q.S. ± 0.01 mm s-1; Line width: Γ ± 0.01 mm s-1; Spectral contribution: ± 3%; *Average magnetic field; a,bFerrihydrite structures with different crystallinity degrees.*

# Solid identification with X-ray diffraction

*[See enclosed document with XRD spectra]*

# References

1. Hyatt D, Chen GL, LoCascio PF, Land ML, Larimer FW, Hauser LJ. Prodigal: Prokaryotic gene recognition and translation initiation site identification. BMC Bioinformatics [Internet]. 2010 Mar 8 [cited 2022 Aug 16];11(1):1–11. Available from: https://bmcbioinformatics.biomedcentral.com/articles/10.1186/1471-2105-11-119

2. Kanehisa M, Sato Y, Morishima K. BlastKOALA and GhostKOALA: KEGG Tools for Functional Characterization of Genome and Metagenome Sequences. J Mol Biol. 2016 Feb 22;428(4):726–31.

3. Tatusova T, Dicuccio M, Badretdin A, Chetvernin V, Nawrocki EP, Zaslavsky L, et al. NCBI prokaryotic genome annotation pipeline. Nucleic Acids Res [Internet]. 2016 Aug 19 [cited 2022 Aug 16];44(14):6614–24. Available from: https://pubmed-ncbi-nlm-nih-gov.tudelft.idm.oclc.org/27342282/

4. Garber AI, Nealson KH, Okamoto A, McAllister SM, Chan CS, Barco RA, et al. FeGenie: A Comprehensive Tool for the Identification of Iron Genes and Iron Gene Neighborhoods in Genome and Metagenome Assemblies. Front Microbiol [Internet]. 2020;11:37. Available from: https://github.com/Arkadiy-Garber/FeGenie.

5. Rost B. Twilight zone of protein sequence alignments. Protein Eng Des Sel [Internet]. 1999 Feb 1 [cited 2022 Jun 6];12(2):85–94. Available from: https://academic-oup-com.tudelft.idm.oclc.org/peds/article/12/2/85/1550637

6. Klencsár Z. Mössbauer spectrum analysis by Evolution Algorithm. Nucl Instruments Methods Phys Res Sect B Beam Interact with Mater Atoms. 1997;129(4):527–33.
